# Designing and conducting initial application of a performance assessment model for in-hospital trauma care

**DOI:** 10.1186/s12913-022-07578-2

**Published:** 2022-03-01

**Authors:** Yalda Mousazadeh, Homayoun Sadeghi-Bazargani, Ali Janati, Mahboub Pouraghaei, Farzad Rahmani, Mobin sokhanvar

**Affiliations:** 1 Department of Public Health, Khalkhal University of Medical Sciences, Khalkhal, Iran; 2 Iranian Center of Excellence in Health Management, School of Management and Medical Informatics, Tabriz University of Medical Sciences, Tabriz, Iran; 3 Road Traffic Injury Research Center, Tabriz University of Medical Sciences, Tabriz, Iran; 4 Department of Health Policy and Management, Iranian Center of Excellence in Health Management, School of Management and Medical Informatics, Tabriz University of Medical Sciences, Tabriz, Iran; 5 Emergency Medicine Research Team, School of Medicine, Tabriz University of Medical Sciences, Tabriz, Iran; 6 Department of Public Health, Khoy University of Medical Sciences, Khoy, Iran

**Keywords:** Performance assessment, Hospital, Traffic injury, Trauma care, Trauma center

## Abstract

**Background:**

Trauma is a major cause of death worldwide, especially in Low and Middle-Income Countries (LMIC). The increase in health care costs and the differences in the quality of provided services indicates the need for trauma care evaluation. This study was done to develop and use a performance assessment model for in-hospital trauma care focusing on traffic injures.

**Methods:**

This multi-method study was conducted in three main phases of determining indicators, model development, and model application. Trauma care performance indicators were extracted through literature review and confirmed using a two-round Delphi survey and experts’ perspectives. Two focus group discussions and 16 semi-structured interviews were conducted to design the prototype. In the next step, components and the final form of the model were confirmed following pre-determined factors, including importance and necessity, simplicity, clarity, and relevance. Finally, the model was tested by applying it in a trauma center.

**Results:**

A total of 50 trauma care indicators were approved after reviewing the literature and obtaining the experts’ views. The final model consisted of six components of assessment level, teams, methods, scheduling, frequency, and data source. The model application revealed problems of a selected trauma center in terms of information recording, patient deposition, some clinical services, waiting time for deposit, recording medical errors and complications, patient follow-up, and patient satisfaction.

**Conclusion:**

Performance assessment with an appropriate model can identify deficiencies and failures of services provided in trauma centers. Understanding the current situation is one of the main requirements for designing any quality improvement programs.

## Background

Trauma is one of the major causes of death worldwide, occurring mostly in the first four decades of life [1]. It is reported that nearly 5.8 million people die each year as a result of trauma [2]. Moreover, trauma occurs in all countries and is a common problem in modern societies [3]. On the other hand, a significant share of the burden of diseases is due to trauma [2, 4]. Also, trauma annually leads to more than 50 million Disability-Adjusted Life Years (DALY) [5]. The other point is that traumatic injuries can cause higher mortality rates than Acquired Immune Deficiency Syndrome (AIDS), Malaria, and Tuberculosis [6]. Therefore, trauma is a critical and time-dependent health issue that requires an instant healthcare intervention to reduce the probability of mortality and disability [7].

Among the various types of trauma, trauma caused by a crash is a life-threatening condition for all age groups [8]. Traffic injuries are reported to have increased from 999,000 in 1990 to more than one million deaths in 2002, and were predicted to reach about two million deaths by 2020 [9]. Evidence shows that the trauma injuries from collisions leads to the death of 21 million people and the disability of 20-50 million people annually, most of whom are young [10, 11]. Traffic injuries are the second leading cause of death in Iran, accounting for 40% of unusual deaths [12, 13]. It is undeniable that the injuries caused by trauma are more severe in LMIC due to the lack of an organized trauma system and the extent of occasions leading to trauma, such as collisions [14].

In the case of trauma care, health organizations are responsible for providing cost-effective, patient-oriented, and safe health services to affected patients at the right time and place [3]. The key feature of good trauma care is rapid transfer to a medical center where appropriate trauma care is available and definitive treatment can be delivered within the first hour after injury [15]. Prevention activities, communication infrastructure, medical direction, trained workforce, pre-hospital care, transportation services, triage, in-hospital care, rehabilitation, public education, and trauma capacity assessment are key components of the trauma care system [15]. Reports indicate that people do not receive the same services for crash injuries even in the same environment. Also, evidence suggests that quality services are not always available to them [3]. Therefore, the quality of care provided to injured patients should be evaluated and improved [16, 17]. Furthermore, the high and rising costs of health services confirm this need [18].

The Committee on Trauma of the American College of Surgeons was one of the first organizations to develop indicators for evaluating trauma care as part of a quality improvement program [19]. However, many LMIC do not have accreditation processes, standards, and specific assessment tools for trauma centers [20]. On the other hand, related indicators have been observed in developed countries for a long time. However, their use in low- and middle-income countries is limited due to resources shortage [21]. In addition, it is recommended to use context-related audit filters in the area of health services provision [5]. Nevertheless, the Iranian Ministry of Health and Medical Education (MOHME) defined only five general criteria as indicators of hospital emergency performance, which are not specifically related to trauma services. Hence, the present study was carried out to design and conduct the initial application of a model for assessing trauma care with a specific focus on traffic injuries.

## Method

This multi-method study was conducted between June 22, 2018 and October 22, 2019 in three main phases of determining the indicators, model development, and model application in the hospital.

### Determining indicators

A comprehensive literature review was conducted by searching five electronic databases, including PubMed, Ovid Medline, Science Direct, ProQuest, Scopus, and Google Scholar search engine. In addition, Persian databases, including Scientific Information Database (SID) and Magiran, were searched. In the next step, the indicators regarding the assessment of in-hospital trauma services were extracted from related articles. Then, the the indicators extracted by a five-member panel were evaluated in terms of feasibility, importance, relationship with the health system, and compliance with context of the Iranian hospitals. Finally, the selected indicators were categorized by content and examined for content validity through a two-round Delphi survey. Accordingly, the Content Validity Index (CVI), Content Validity Ratio (CVR), and the modified kappa were calculated [22, 23]. Participants (*n* = 30) consisted of 17 physicians and specialists (general practitioner, emergency medicine, anesthesiologist, orthopedics, internists, and neurologist), four nurses, and nine faculty members on health policy, disaster and emergency health, and healthcare management. All the participants had work or research experience in trauma care.

The proposed indicators were rated using a self-administered questionnaire. In the first round of the Delphi survey, the questionnaires were distributed after providing sufficient explanations and a deadline of 2 weeks was set for their completion. In order to determine CVI, participants were asked to score each indicator, separately, in terms of their opinions and knowledge relating to three respects, “relevancy to the subject,” “simplicity” and “clarity” using a four-point Likert scale (completely relevant, relevant, relatively relevant, and not relevant). In order to determine CVR, participants were scored each indicator, in terms of “necessity” (necessary, useful but not necessary, and not necessary). They were also asked to comment on the proposed indicators. The formula to calculate CVI and CVR were as follows [23]:

$$\mathrm{CVI}=\frac{\mathrm{Number}\kern0.5em \mathrm{of}\kern0.5em \mathrm{raters}\kern0.5em \mathrm{giving}\kern0.5em \mathrm{a}\kern0.5em \mathrm{rating}\kern0.5em 3\kern0.5em \mathrm{or}\kern0.5em 4}{\mathrm{Table}\kern0.5em \mathrm{number}\kern0.5em \mathrm{of}\kern0.5em \mathrm{raters}}$$

$$\mathrm{CVR}=\frac{\left[\mathrm{n}\hbox{-} \left(\frac{\mathrm{N}}{2}\right)\right]}{\frac{\mathrm{N}}{2}}$$

In terms of CVI, each item with a score higher than 79% is appropriate. Items between 70 and 79% need to be corrected and less than 70% unacceptable. The acceptable CVR was 0.33 according to the number of participants (30 people).

To calculate the modified Kappa, the odds ratio of the agreement was first calculated. In this regard, a binomial random variable formula was used:

$${p}_c=\left[\frac{N!}{A!\left(N-A\right)!}\right].{5}^N$$

In this formula, N is the number of participants, and A is the number of people who agree (the number of raters giving a rating of 3 or 4). In the next step, the Kappa coefficient was calculated based on the following formula:

$$\frac{I- CVI-{P}_C}{1-{P}_C}$$

Based on the Polit and Beck view [23], the kappa coefficient in the range of 0.40-0.75 was at a good level, and above 0.75 was at a high level. The second round presented the indicators based on CVI scores, CVR, and the modified kappa. Finally, the approved indicators were listed and categorized in the second round.

### Model development

Two Focus Group Discussions (FGD) were held (a total of 12 people in two sessions), and 16 semi-structured interviews were performed to identify how trauma care was assessed using the using extracted indicators. Each FGD and interview lasted 60 to 90 and 45 to 60 min, respectively. The participants were selected based on purposive sampling [24]. After obtaining informed consent, the participants’ statements were electronically recorded and then transcribed verbatim. The content analysis method [25] was used to analyze the text of interviews and FGDs. Eighty percent of the participants in this phase were related to the previous stage (Delphi and Panel). According to FGDs and interviews, the initial form of the model and its components were developed.

A three-part questionnaire was designed and provided to 10 selected experts in a separate session to conduct model approval. The majority of these people were faculty members and also worked in trauma centers. The first part consisted of socio-demographic variables of ten participants (Table 1). In the second part, the opinions of experts about the main and sub-components of the model were questioned according to the criteria of importance and necessity, simplicity, clarity, and relevance, based on the 9-point Likert scale. The components with scores between 7 and 9 were approvedand components with scores between 1 and 3 were removed. Agreement on components with a score of 4 to 6 was also discussed [26]. The third part of the questionnaire included questions for validation and agreement on the final form of the model. Accordingly, 12 areas were examined. These 12 areas included model feasibility, compatibility with upstream documents, acceptance of the proposed model by stakeholders, efficiency, flexibility, model sequence, model fit, the balance between model components, and a general question. According to these 12 items, a form was designed based on the 4-point Likert scale (1: very low, 2: low, 3: high, 4: very high), and experts expressed their opinions. Then, the Item-level Content Validity Index (I-CVI) and KAPPA were obtained [27].

**Table 1:** The participants’ characteristics in specialized meetings to review the model components

I	Education	Job	Age^**b**^	Work experience^**c**^
1	Healthcare management	Faculty member	55	25
2	Health policy	Faculty member	30	2
3	Neurologist	Faculty member	52	25
4	General practitioner	Head of the provincial health center	51	23
5	Nurse	Assessor of treatment deputy in university	50	29
6	Emergency Medicine	Faculty member	43	8
7	General practitioner	Researcher of RAPRC^a^	52	21
8	Emergency Medicine	Faculty member	39	8
9	Nurse	Faculty member	52	24
10	Anesthesiologist	Faculty member	45	10

^a^Road Accident Prevention Research Center, ^b, c^Numbers by the year

*Ph.D.* Doctor of Philosophy; *MD *Medical Doctor

### Model application

The initial model application was conducted to assess in-hospital trauma care at the trauma center in the metropolitan city of Tabriz in the East Azerbaijan Province, Iran. Units involved in providing the data needed for general indicators included the medical record department, the emergency medicine department, the quality improvement office, the patient safety office, the nursing office, and the trauma ward. It should be noted that information on general indicators was collected for 1 year.

To collect data on specific indicators, 200 patients were selected who were to the referral trauma center in the emergency department for 3 months. A checklist consisting of 27 questions was designed based on the consensus of the research team and two emergency medicine specialists. Four other experts commented on the checklist, and thus, its validity was confirmed. Cronbach’s alpha coefficient for the four dimensions of the checklist was calculated to be 0.7, 0.65, 0.73, and 0.71, respectively. The checklist consisted of patient information, crash mechanism, patient triage level, vital signs, Glasgow Coma Scale (GCS), some procedures performed for the patient including (intubating, setting chest tube, fracture fixation, bleeding control, and blood and fluids transfusion), diagnostic procedures and related waiting time, patient satisfaction, and the final decision in the emergency department.

**Fig. 1:**
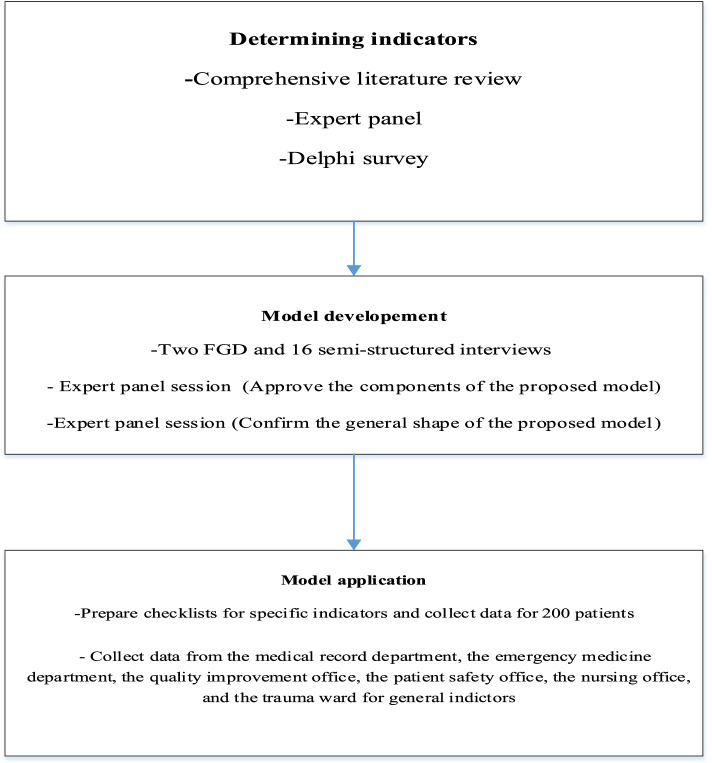
Flowchart of study procedure

The data were collected using patient observation and measures taken to provide services, and interviewing patients and their accompanies, asking staff, and reviewing the patient's document. The gathered data were analyzed using Stata software (Stata 14 package statistical software). Then, the data were reported using descriptive statistics. One-Sample Kolmogorov-Smirnov Test was applied to test the normality of the distributions of the variables. Accordingly, Kruskal-Wallis, Chi-square, One Way ANOVA, and linear regression tests were also used to examine the relationship between variables. Figure 1 gives an overview of the study procedures.

## Results

### Determining indicators

**Fig. 2:**
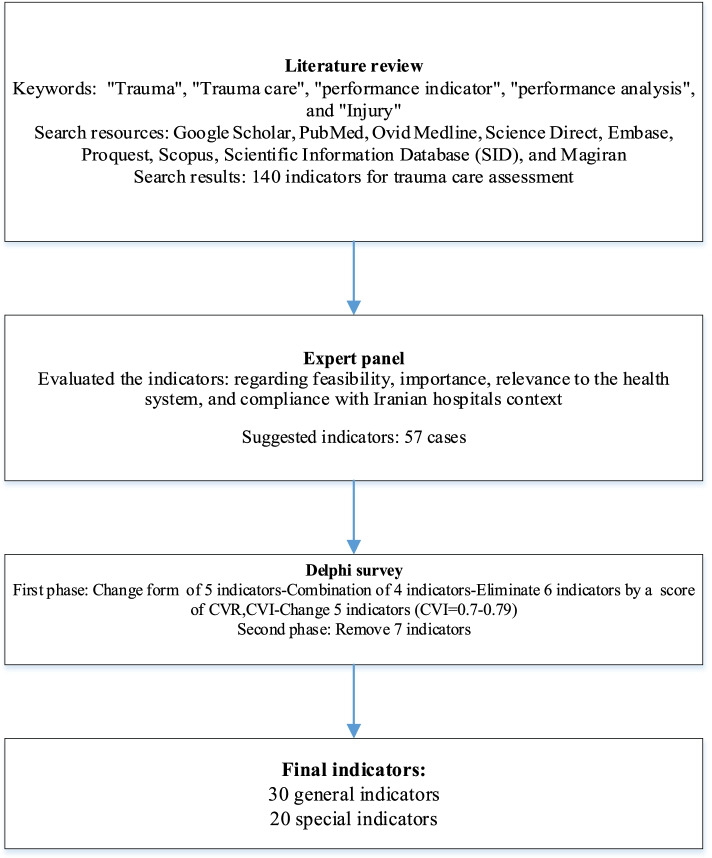
Flowchart of determining indicators

**Fig. 3:**
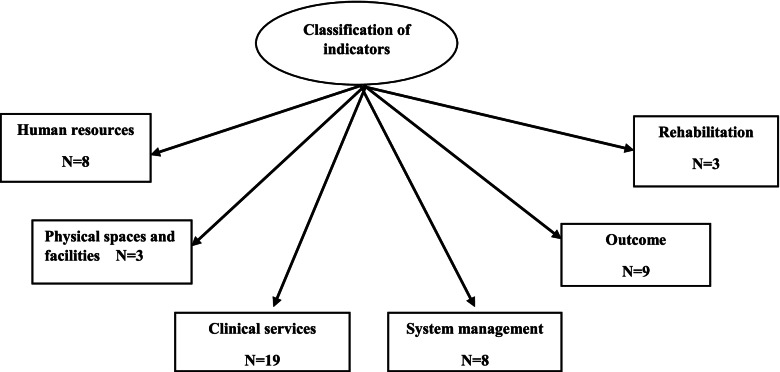
Classification of trauma care indicators for assessment

In a comprehensive literature review, 140 indicators were associated with in-hospital trauma care after reviewing 51 articles, 3 guidelines, and 2 books. Then, some indicators were excluded or merged due to insignificance, differences in the management system of countries, lack of sufficient data (physical and electronic), time, human and physical resources shortage. Therefore, in the Delphi survey, 57 indicators were entered and investigated. In the first phase, CVR, CVI, and Modified Kappa were calculated to be 0.64, 0.85, 0.83, respectively. A total of 50 indicators were confirmed in the second phase of Delphi. Figure 2 provides an overview of this step of the study. Also, classification indicators for assessing in-hospital trauma care are shown in Fig. 3.

### Model development

**Fig. 4:**
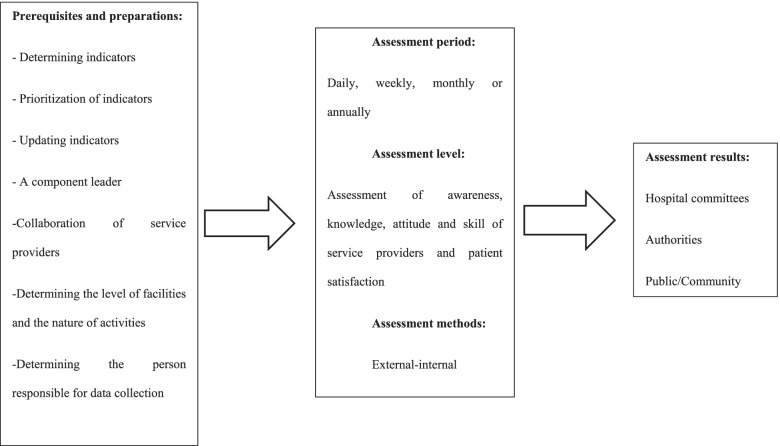
The FGDs and interviews finding

Prerequisites and assessment steps were determined based on expert opinions. Accordingly, assessment prerequisites included a component leader, support of service providers, determining the level of facilities and the nature of activities, and a person to collect data. The specialists insisted on constant review of indicators and updating if necessary. This was because the indicators had to be evidence-based and relevant to the results. Determining the assessment period (daily, weekly, monthly or annual) was another necessity. The experts believed that assessment should have broad dimensions and different aspects, including assessment of awareness, knowledge, attitude, service providers’ skill, and patient satisfaction. The experts asserted that both external assessments by auditing organizations and internal assessments by process owners should be considered. Finally, the experts suggested that the results of the evaluation be reviewed by hospital committees and relevant authorities. Then be made public. Figure 4 provides an overview of the FGDs and interviews findings.

Based on the expert opinions, the initial form of the performance assessment model was designed in six components by the research team. These components were:

1. Selection of the assessment level (including hospital wards and patient/ staff views)
2. Assessment team (specialized and non-specialized)
3. Measurement method (assessment content based on the Donabedian framework Measuring tool)
4. Scheduling (based on plan or case)
5. Frequency of assessment (general and specific)
6. Data source (current reports, periodic reports, and case reports)

**Fig. 5:**
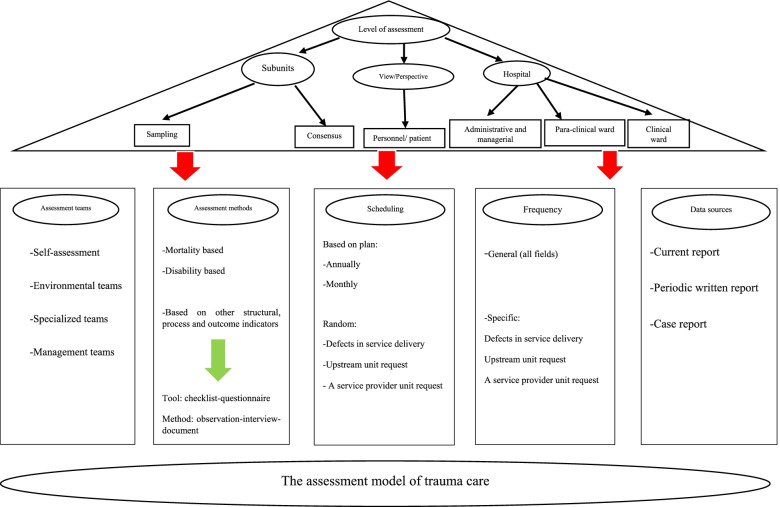
Assessment Model for in-Hospital Trauma Car

All components of the model scored an average of 7 to 9 in the Delphi survey and were therefore confirmed. The experts agreed on all the components as well as the general shape of the proposed model. The self-assessment team was added to the assessment teams based on their comments. Also, the k-score and I-ICV was obtained as 1 except for two areas of the model, including acceptability by stakeholders and the model’s simplicity in other areas under study. Figure 5 shows the final form of the model.

### Model application

A total of 5163 crash patients were transported by ambulance in 1 year. The number of patients admitted to the emergency department was 330-340 patients per day. A total of 1951 patients were hospitalized, which was 0.04% of the total hospitalized patients. A trauma registry related to collisions was set up in the trauma center. The in-hospital trauma team consisted of emergency medicine specialists and senior residents in surgery, internal medicine, orthopedics, and neurologists. Eleven Root Cause Analyses (RCA) were performed over 1 year, none of which was for crash patients. Information related to Failure Modes and Effects Analysis (FMEA) was not recorded. The level of satisfaction of trauma patients referred to the emergency department was not assessed during a year. The patient’s functional status was not assessed after discharge, and there was no protocol for referral to rehabilitation centers. Table 2 shows the results of other general indicators.

**Table 2:** Results of assessing general indicators

Indicator	Results (Frequency or percent)	Description
The average length of stay of trauma victims (day)	5.8	Statistics related to crash patients were not available separately, and the result was related to the trauma ward. The length of stay in ICU was 7.25 days.
The ratio of full-time emergency physicians to the number of patients	0.01	For every 55 patients, there was one emergency medicine specialist per shift.
The ratio of full-time emergency assistants to the number of patients	–	The number of assistants per shift varied.
The ratio of nurses to the number of patients in the emergency department	0.13	There was one nurse for every seven patients. Adequacy confirmation required more assessment.
The ratio of patient carriers to the number of patients in the emergency department	0.12	There was one patient carrier for every eight patients. Adequacyconfirmation required more assessment.
The ratio of cleaner staff to the number of patients in the emergency department	0.06	There was one cleaner staff for every 15 patients. Adequacyconfirmation required more assessment.
The percentage of physicians working in the trauma emergency department who have completed ATLS and received a valid certificate	100%	–
The percentage of trauma nurses who have completed ATCN and received a valid certificate	84.05%	All nurses must take the required courses.
The ratio of resources required to manage airway, breathing, circulation, and shock (based on the WHO checklist)	100%	–
The ratio of specific resources for special injuries management including head, neck, chest, abdomen, extremity, spinal, burns, and wounds (Based on the WHO checklist)	84.05%	Auto transfusion from chest tubes and topical antibiotic dressing were not adequate. Also, maintaining norm tension and oxygenation to prevent secondary brain injury, portable X-ray was not existence.
Tonometer for proper treatment of compartment syndrome	0	
Per capita physical space of the emergency department in proportion to the number of patients (daily on average)	5.4 square meters per patient in each shift	The physical space of the emergency room was 1815 square meters.
The number of death-related audits based on the latest version of ICD	3	Three of the eight deaths were related to crash patients’ mortality audits.
The number of occurred errors	Bedsore (45) Medication errors (40) Registration errors (36) Other cases (19)	The recorded errors were 180 cases, and they only were related to nurses’ errors.
The number of sessions held to examine the deaths of traumatic victims and the number of approvals implemented based on it	3	Out of 50 approved approvals, three were related to crash patients in a year.
The number of quality improvement sessions to examine the problems related to providing service for traumatic patients and the number of approvals implemented based on it	0	–
The percentage of patients dispositioned in less than 6 h	94.09%	The average time out of the emergency room was about four hours.
The percentage of successful CPR in traumatic patients	1.19%	This index was not calculated for eight months. It was not calculated separately for collisions
The mortality rate of traumatic patients	10.45%	A total of 2124 road traffic patients were admitted to the hospital, of which 222 patients died.
The number of the incidence of hospital complications	6	Only postoperative infection and bleeding were recorded.
The percentage of the visit or re-hospitalization in the emergency department	16.64%	The number of road traffic hospitalizations was 4228, and 707 were readmitted. The cause of readmission is not specified.
The ratio of crash mortality to the number of dead patients	9.11%	The total number of fatalities was 2435, and the number of fatalities due to accidents was 222 (in a year).

[3]

*ATLS* the Advanced Trauma Life Support course; *ATCN* the Advanced Trauma Care for Nurses course; *ICD* International Classification of Diseases; *CPR* Cardiopulmonary Resuscitation; *ICU* Incentive Care Unit; *WHO* World Health Organization

In this study, 200 crash patients referred to the hospital emergency department were examined. The mean and Standard Deviation (SD) of patients’ age were 33.13 and 19.04, respectively. 42% of patients were sent to the hospital from different cities of East Azerbaijan province and 58% from Tabriz city. The mean (SD) of GCS in 170 patients was 14.41 (2.36). Out of 200 patients, imaging services were performed for 186 patients (93.47%). Table 3 shows some demographic characteristics and hospital information of the patients. Table 4 shows the waiting time for Para clinical procedures and patient outcomes in the emergency department.

**Table 3:** Demographic characteristics and hospital information of the patients

Variable	Frequency (%)
Gender	
Male	161(80.5)
Female	39(19.5)
Mechanism	
The collision of vehicles with pedestrian	100(50)
The collision of vehicles with each other	42(21)
The collision of the vehicle with a motorcycle	25(12.5)
The vehicle and motorcycle overturning	15(7.5)
Other	18 (9)
Location of injury	
Head and face	67 (33.5)
Arms and hands	33(16.5)
Neck	19(9.5)
Chest and abdomen	3(1.5)
Posterior trunk (the back and spine)	5(2.5)
Pelvis	7(3.5)
General weakness	10(5)
Multiple trauma	44(22)
Triage level	
Level 1	12(6.06)
Level 2	44(22.22)
Level 3	142(71.72)
Documentation	
Completeness of the patient document	87(43.5)
T record	91(45.5)
SO2 record	43(21.5)
PR record	14(7)
RR record	43(21.5)
BP record	20(10)
GCS record	170(85)
Some clinical procedures	
Pulse oximetry	182(91)
Chest tube	6(3)
Intubation	5(2.5)
Muscular skeletal checking	188(94)
DPL	3(1.5)
Fracture fixation	29(14.5)
Blood transfusions and blood products	20(10)
Outpatient surgery	62(31)
Heparin and enoxaparin injection	4(2)
Outcome	
Discharge with medical advice	104(52)
Discharge against medical advice	9(4.5)
Escape	2(1)
Dispatch to another medical center	56(28)
Hospitalization in inpatient wards	27(13.5)
Hospitalization in incentive unit care	2(1)
Paraclinical services	
CT scan	59(31.72)
CXR	67(36.02)
FAST	115(61.82)
Abdominal and pelvic ultrasound	2(1.07)
ECG	5(2.68)

*T* Temperature, *SO2* Saturation of Oxygen, *PR* Prothrombin Ratio, *RR* Respiratory Rate, *BP* Blood Pressure, *GCS* Glasgow Coma Scale, *DPL* Diagnostic peritoneal lavage, *CT* Computerized tomography, *CXR* Chest X-Ray, *FAST* Focused Assessment with Sonography for Trauma, *ECG* Electrocardiogram

**Table 4:** Waiting time of preclinical services and outcomes

Service	Median (minute)	Min (minute)	Max (minute)
Paraclinical services			
CT	54	4	324
CXR	36	4	280
FAST	35	6	195
Sonography	32.5	6	55
ECG	30	10	135
Outcome			
Discharge	170	21	1111
Dispatch to another medical center	151.5	50	720
Hospitalization in normal wards	189	10	1709
Hospitalization in special wards	720	240	1200

*CT* Computerized Tomography; *CXR* Chest X-Ray; *FAST* Focused Assessment with Sonography for Trauma; *ECG* Electrocardiogram

The Chi-square test showed a statistically significant relationship between outcome and mechanism of injury, location of injury, and GCS group (*p* < 0.05). Based on Kruskal–Wallis test, the relationship between the GCS score and the outcome was statistically significant (*p* < 0.05). There was a statistically significant relationship between triage level and outcome (*p* < 0.05). The waiting time to receive a CT scan increased the waiting time for discharge by 1.46 times (*p* < 0.05). Also, the waiting time for receiving CXR increased the waiting time for discharge by 1.56 times (*p* < 0.05).

Out of 200 patients, two patients were not triaged, or their triage was not recorded. In addition, five patients were re-triaged due to lower triage than the injury level of the patients. The neuromuscular status of 12 patients was not checked. There was no vacancy for treatment for eight patients in the operating room at the time of the medicines order. The decision was delayed in five patients due to a malfunction due to the failure of the Picture Archive and Communication System (PACS). There was a delay in depositing five patients due to receiving counseling. The hospitalization of five patients was delayed due to a lack of empty beds. Table 5 shows the quality of some of the procedures performed for patients.

**Table 5:** The quality of some of the procedures performed on the patient

Item		Likert degree Frequency (%)				
		Very good	Good	Median	Poor	Very poor
Chest tube						
1	Selecting the correct cutting location and tube size		1(16.67)	5(83.83)		
2	Identifying the location of the tube		4(66.67)	2(33.33)		
3	Inserting the tube		4(66.67)	2(33.33)		
4	Fixation		5(83.83)	1(16.67)		
5	Functional check		4(66.67)	1(16.67)	1(16.67)	
Intubation						
1	Providing equipment, laryngoscope checks, and medications, the appropriate size of the endotracheal tube		1(20)	4(80)		
2	The correct way to get an ambo bag		5(100)			
3	Correct drug injection sequence		5(100)			
4	Appropriate laryngoscopy		5(100)			
5	Proper tube placement		4(80)	1(20)		
6	Endotracheal tube fixation and proper lung ventilation check		5(100)			
Blood and fluid transfusions						
1	Checking patient characteristic		20(100)			
2	Checking the blood product and patient’s blood type		20(100)			
3	Matching delivered blood type and patient blood type		20(100)			
4	Recording the date and duration of the injection		19(95)	1(5)		
Splinting						
1	Providing wound cleansers (if available)		14(48.28)	13(44.83)	2(6.9)	
2	Suitable analgesia for the patient		15(51.72)	12(41.38)	2(6.9)	
3	The right size splint		23(79.31)	3(10.34)	3(10.34)	
4	Proper installation (observing the top and bottom of the splint - how to get the limb)		21(72.41)	2(6.9)	6(20.69)	
5	Limb pulse check after implantation		20(68.97)	2(6.9)	7(24.14)	
Patient satisfaction						
1	waiting time		152(76)	3(1.5)	45(22.5)	
2	Physician skills and behavior		182(91)		18(9)	
3	Nurse skills and behavior		187(93.5)		13(6.5)	
4	Supplies and equipment		188(94)		12(6)	

## Discussion

This is a unique study from Iran in the field of trauma care. In this study, in-hospital trauma care indicators were extracted, and a model was developed to assess hospital performance in the management of trauma patients. Finally, a field study was firstconducted to apply the model in a trauma center, which contained positive findings and, of course, the shortcomings and weaknesses that were discussed.

A total of 50 indicators related to trauma care were identified and presented to be applicable in the Iranian hospital context. Indicators are used to compare the current situation with the standards, identify defects and shortcomings and fix them to improve performance [18, 28]. Of course, the usefulness of using indicators is when they are compatible with the requirements of the context [28]. So, in this study, among the indicators extracted from the literature review, those were selected which fit the Iranian hospitals’ conditions based on the expert opinions. Evidence suggests that context-based filters will be useful. For example, the failure to use trauma care indicators in Asian countries has been due to a lack of standard data collection mechanisms and limited resources [5].

In this study, a six-dimensional model was also designed to assess the performance of in-hospital trauma care based on the expert opinions. The use of international indicators and models is essential in the event of localization, especially in developing countries that do not have an accreditation process for trauma centers [5, 20]. Moeini et al. used the survival probability assessment system and concluded that despite the differences between the developed model of the countries as well as Iran; this model could be useful after localization and development of coefficients and variables derived from regional databases [29].

Experts in this study focused on important and applicable indicators due to the limitations of LMIC. Studies show that due to resource deficiencies, the use of low-cost interventions is more beneficial by increasing the efficiency and quality of care [30, 31]*.* Studies in Iran and Pakistan have focused on improving trauma care processes to prevent death and disability even in low-resource centers [32, 33]. In addition to the lack of resources, simpler indicators that can be collected based on current data allow for more comparisons. However, in hospitals with more resources, more advanced indicators can be used [5]. It is difficult to define the best performance in trauma, which is related to lack of financial resources and organizational problems such as the unclear definition of responsibilities within trauma teams and resistance to clinical guidelines. Therefore, in centers with the same level of resources, there is a difference in the quality of service [34]. These studies show that emphasizing the effectiveness of routine processes in evaluations is useful and allows comparisons that were also considered in designing the model of the present study.

Receiving the opinions of service recipients as an evaluation level was one of the strengths and innovations of the model presented in this study. Murray points out that the issue of quality has been raised from the service recipients in various health programs to continue to use care, ensure effectiveness, and participation of peopleand other stakeholders in health care [35]. Santana et al. also stated that a comprehensive, patient-centered assessment is achieved when patient perspectives are considered [36]. Simultaneous attention to structure, process, and outcomes is another strength of the model presented in this study, which is recommended by Donabedian [28].

Mortality was one of the leadings used indicators in this study which is a key indicator in assessing trauma care [19]. Findings showed that the mortality rate of crash patients was about 9% over a year. In Iran, the prevalence of collisions is twenty times higher than the global average [37]. Furthermore, the World Road Safety report revealed that the death rate due to crashs in Iran is estimated to be 32.1 per 100,000 people [38]. Disability was another indicator used in the current study. Unfortunately, the pilot assessment in a trauma center revealed that the functional status of the patients is not evaluated before discharge. This is while the findings of studies have shown that patients rarely have a definite and stable state of health at the time of discharge [39, 40].

Assessment findings also showed that many quality improvements measures, including the death audit and comprehensive recording of errors and complications, RCA and FMEA at the study trauma center were in poor condition. While, in their review, authors concluded that most quality improvement measures could be useful in trauma care [41]. Suitable efforts to improve quality and support patient safety can improve patient-based outcomes and reduce costs [42].

Another important finding of the assessment was that most injured were pedestrians. In line with the present study, the classification of the patients injured in collisions in Iran showed that pedestrians with 39.8% had the most injuries [43]. Among different parts of the body, the head was the most frequently injured part in this study. In line with this finding, Taghipour et al. stated that head trauma had been the most frequent injury in driving accidents [44].

The length of stay in the emergency department was another investigated indicator which was very long in studied center. This is important because 75-85% of deaths occur in the first 20 min after an accident [45]. Also, in patient safety measures, results showed that only two cases of complications were studied, including postoperative infection and postoperative bleeding. This is while there were six cases of surgical site infection during the data collection period. Surgical site infections, with an incidence of 2 to 5%, account for 24% of nosocomial infections and increase morbidity and mortality [46].

Error reporting in the study center was not based on a precise mechanism, and most reports were provided by nurses. The most common errors reported were included registration errors and medication mistakes. As in the present study, findings reported by Bozorgzad and Hemmat showed that most of the reported errors were related to nurses [47]. Jolaee et al. reported medication mistakes as the most common error, consistent with findings of the present study [48].

Regarding the factors associated with patient satisfaction, the findings of this study showed that waiting time was an important factor in this regard. In line with this finding, in a study of physicians’ opinions working in the emergency department about the cause of patients’ dissatisfaction, 61% of them stated that waiting time is the main cause [49].

Finally, the initial application of the designed model revealed deficiencies in some of the equipment needed for trauma care. Lack of facilities to provide trauma services and shortage of related equipment in many developing countries is evident, as Mook et al. have concluded in the case of Mexico, Vietnam, Ghana, and India [50].

### Limitations and strength

This study is the first to design an in-hospital trauma care assessment model by combining scientific evidence with expert opinions to be more in line with the conditions of Iranian hospitals. The development of this model is the first step in a series of research studies designed to improve our understanding and improve measurement and assessment of hospitals’ performance in terms of trauma care. However, this study also had some limitations. While every attempt was made to comprehensively search the literature. Indicators may have been excluded due to the impossibility of collecting data and as a result theindicators of in-hospital trauma care that may not have been examined. Initial application of the model in a trauma center is another potential limitation of the study. To validate the model, it is necessary to apply it in more centers.

## Conclusion

The indicators and the steps required to assess the performance of in-hospital trauma care were designed based on a literature review and experts’ opinions and presented in the form of a model. The use of the model leads to a systematic assessment and comprehensive review of performance. Model application revealed some problems regarding information registration, complications recording, patient satisfaction, improper clinical services, follow-up and rehabilitation services, and waiting time. Identifying performance deficiencies may lead to appropriate planning and performing the necessary interventions. So, it can be expected to improve the outcomes of patients, hospitals, and the health system.

## Data Availability

The datasets used and analyzed during the current study are available from the corresponding author on request.

## Abbreviations

DALY: Disability-Adjusted Life Years
AIDS: Acquired Immune Deficiency Syndrome
MOHME: Ministry of Health and Medical Education
CVI: Content Validity Index
CVR: Content Validity Ratio
FGD: Focus Group Discussion
I-CVI: Item-level Content Validity Index
RCA: Root Cause Analyses
FMEA: Failure Modes and Effects Analysis
SD: Standard Deviation
PACS: Picture Archive and Aommunication System
PhD: Doctor of Philosophy
MD: Medical Doctor
ATLS: the Advanced Trauma Life Support
ATCN: the Advanced Trauma Care for Nurses
ICD: International Classification of Diseases
CPR: Cardiopulmonary Resuscitation
ICU: Incentive Care Unit
T: Temperature
SO2: Saturation of Oxygen
PR: Prothrombin Ratio
RR: Respiratory Rate
BP: Blood Pressure
GCS: Glasgow Coma Scale
DPL: Diagnostic Peritoneal lavage
CT: Computerized Tomography
CXR: Chest X-ray
FAST: Focused Assessment with Sonography for Trauma
ECG: Electrocardiogram
SID: Scientific Information Database
WHO: World Health Organization
